# A Feasible Laboratory-Strengthening Intervention Yielding a Sustainable Clinical Bacteriology Sector to Support Antimicrobial Stewardship in a Large Referral Hospital in Ethiopia

**DOI:** 10.3389/fpubh.2020.00258

**Published:** 2020-06-23

**Authors:** Cedric P. Yansouni, Daniel Seifu, Michael Libman, Tinsae Alemayehu, Solomon Gizaw, Øystein Haarklau Johansen, Workeabeba Abebe, Wondwossen Amogne, Makeda Semret

**Affiliations:** ^1^J.D. MacLean Centre for Tropical Diseases & Divisions of Infectious Diseases and Medical Microbiology, McGill University Health Centre, Montreal, QC, Canada; ^2^School of Medicine, Addis Ababa University, Addis Ababa, Ethiopia; ^3^Microbiology Laboratory, Tikur Anbessa Specialized Hospital, Addis Ababa, Ethiopia; ^4^Department of Microbiology, Vestfold Hospital Trust, Tønsberg, Norway

**Keywords:** bacteriology, laboratory strengthening, diagnostics, antimicrobial stewardship, Ethiopia, non-malarial febrile illness, sepsis

## Abstract

**Background:** Access to clinical bacteriology in low resource settings (LRS) is a key bottleneck preventing individual patient management of treatable severe infections, detection of antimicrobial resistance (AMR), and implementation of effective stewardship interventions. We sought to demonstrate the feasibility of a practical bundle of interventions aimed at implementing sustainable clinical bacteriology services at Tikur Anbessa Specialized Hospital in Addis Ababa, Ethiopia, and report on cost and intensity of supervision.

**Methods:** Starting in Dec 2015, an intervention based on the CLSI QMS01-A guideline was established, consisting of (i) an initial needs assessment, (ii) development of key standard operating procedures, (iii) adaptation of processes for LRS, (iv) training and supervision of laboratory staff via consultant visits and existing online resources, and (v) implementation of a practical quality systems approach. A guiding principle of the bundle was sustainability of all interventions post implementation.

**Outcomes and challenges:** An initial investment of ~US$ 26,200 for laboratory reagents, and a total of 50 visit-days per year from three Canadian and Norwegian microbiologists were committed. Twelve SOPs, including antimicrobial susceptibility testing, were adapted, and an automated blood culture platform was donated (bioMerieux). In the first 18 months of implementation of the intervention, the average volume of specimens analyzed in the lab went from 15/day to 75/day. The number of blood cultures tested increased from an average of 2/day to over 45/day. Antimicrobial susceptibility testing was introduced and cumulative antibiograms were generated for the institution. Quality control was implemented for all procedures and quality assurance tools implemented included external quality assurance and proficiency testing of six technologists with longitudinal follow-up. The laboratory is on the path toward SLIPTA accreditation by the African Society for Laboratory Medicine. Reagent costs, staff training and retention, and engagement of clinical personnel with the lab proved to be manageable challenges. Key external challenges include in-country supply-chain management issues, lack of competition among distributors, and foreign-currency exchange distortions.

**Conclusions:** Using a relatively low-intensity intervention based on existing training tools and accreditation schemes, we demonstrate that establishment of reasonable-quality clinical bacteriology is not only within reach but also a critical step toward assessing the burden of AMR in settings like this one and implementing effective stewardship strategies.

## Introduction

Access to clinical bacteriology testing in low resource settings (LRS) is a key bottleneck preventing individual patient management of treatable severe infections, detection of antimicrobial resistance (AMR), and implementation of effective antimicrobial stewardship (AMS) interventions. In LRS, 10–15% of all hospitalized patients develop healthcare-associated infections, a major cause of death (1–3). Widespread bacterial resistance to almost all antimicrobials available in these settings exacerbates the difficulties of selecting empiric therapy. This reality, coupled with the importance of identifying hospitalized patients in whom antibiotics are *not* required, has prompted the general recognition that bacteriology testing in LRS must be prioritized (4). Yet, the shortcomings of diagnostic laboratories in LRS and their negative impact on clinical care and control of infectious disease outbreaks are well-described (5–8), with the greatest gaps seen in sub-Saharan Africa. Strengthening laboratory network capacity has become a focus of international efforts in the last decade, with initiatives aimed at surveillance (9), health system strengthening and accreditation (10–14), and global health security (15). In 2018, availability of on-site bacteriology infrastructure was declared a requirement for secondary-level hospitals in the WHO Model Essential Diagnostics List (16).

Unfortunately, among ~500 medical laboratories accredited to international standards in sub-Saharan Africa, almost none are clinical hospital laboratories that provide bacteriology diagnostics (13). Recognizing the gulf between high-level recommendations and the current availability of clinical bacteriology laboratories in sub-Saharan Africa, we sought to demonstrate the feasibility of a practical bundle of interventions aimed at implementing sustainable clinical bacteriology services at Tikur Anbessa Specialized Hospital in Addis Ababa, Ethiopia, and report on cost, laboratory utilization, and solutions to problems encountered.

## Context

Ethiopia is the second most populous country in sub-Saharan Africa and also among its poorest, with a per capita income of US$790 in 2019 (17). However, it has the fastest growing economy in the region and aims to reach lower-middle-income status by 2025 (17). At the start of our study in December 2015, there were no accredited bacteriology laboratories and little access to bacteriology testing of any kind in Ethiopia. The Tikur Anbessa Specialized Hospital (TASH) in Addis Ababa is the largest referral hospital in the country, with 800 in-patient beds and approximately 20,000 admissions/year with an average length of stay of 9.3 days. Addis Ababa University, Ethiopia, has a longstanding collaboration with McGill University, Canada, that has yielded an Infectious Diseases subspecialty training programme, research projects, and The Addis Ababa University (AAU) and McGill Partnership for Infectious Diseases (AMP-ID, http://amp-id.org/. The working language in the hospital is English.

## Intervention and Methods

We devised a laboratory-strengthening intervention as the first component of a 4-year funded project which included the laboratory intervention, establishment of antimicrobial stewardship, and surveillance of hospital-acquired infections (RI-MUHC Grant #974). The laboratory intervention itself was established in the first 18 months and these are the data presented here. An initial needs assessment was made in December 2015 to document laboratory personnel and infrastructure, laboratory utilization, and quality assurance measures in place. It revealed a hospital bacteriology laboratory of ~50 m^2^, with 6 full-time technologists and a 50-bottle automated blood culture platform, which had not been used for several years due to lack of reagents. Technologists had Diplomas or Bachelor's degrees in Clinical Laboratory Science/Medical Technology, or MSc in Microbiology, with experience ranging from one to over 10 years. There were no medically qualified or PhD microbiologist supervising the laboratory. Work was organized on a single bench, on which different specimen types were batched separately. Several infrastructural elements were already in place, including a class-2 biosafety cabinet, incubator, refrigerator, and a separate media room. There were very few specimens processed daily (approx. two blood culture requests per day), all processed manually. In bench side assessments of routine work, staff proficiency was highly variable and there were few quality measures in place, such as formal standard operating procedures (SOP) or documentation of quality control of reagents. Unsurprisingly, our visit highlighted the absence of a working relationship between clinicians and the laboratory, fuelling its underperformance and neglect.

A minimal intervention bundle based on the CLSI QMS01-A4 guideline (18) was established, consisting of (i) the initial needs assessment, (ii) development of key SOPs, (iii) adaptation of processes for LRS, (iv) training and supervision of laboratory staff via consultant visits and existing online resources, and (v) implementation of a practical quality systems approach. A guiding principle of the bundle was sustainability of all interventions post implementation.

### Development of Key Standard Operating Procedures (SOP)

We identified a minimum set of 14 SOP to be developed for the TASH microbiology laboratory (Table 1). Bloodstream infections were prioritized because they are associated with the greatest mortality among hospital-acquired infections (19). We used SOP documents in use at the McGill University Health Center as a starting point and adapted these according to the intended audience and local procedures. The overriding principle that guided the adaptation of SOPs was the need for them to be understood and accessible to the technologists in the lab, taking into account level of training and language proficiency. Examples of explicit measures we took included adopting straightforward language, short sentences, a legible font and size, and avoiding acronyms. These principles and best-practices for developing SOP for LRS have been reviewed in detail (20). We were surprised to find that pictographic representations of processes, such as flow-diagrams or illustrations, were consistently misunderstood by the team. These were more complicated to interpret for many technologists than clearly-written English text. Thus, although figures may be required to convey specific messages, they should not be used as a replacement of plain language.

**Table 1:** Standard operating procedures (SOP) that were developed and implemented as part of the initial laboratory strengthening intervention.

Antimicrobial susceptibility testing using Kirby Bauer disc diffusion method*
Blood bacterial culture
Cerebrospinal fluid bacterial culture*
Sterile body fluid bacterial culture
Pus—superficial specimens—bacterial culture
Pus—deep specimens—bacterial culture
Urine bacterial culture
Sputum and lower respiratory specimen bacterial culture
Vaginal bacterial culture
Gram stain
Identification of Gram-negative bacilli
Identification of Gram-positive cocci
Identification of Gram-positive bacilli
Recognizing dangerous pathogens at the work bench

*This table does not list SOP dealing with quality control*.

* *These documents are provided in the Supplemental Appendix for illustrative purposes*.

### Adaptation of Analytic-Phase Processes for This Setting

Bacterial culture aims to rule-in the presence of pathogenic bacteria, identify them, and determine their antimicrobial susceptibility. The most important reason for identifying bacteria is to allow the application of appropriate antimicrobial susceptibility breakpoints. Since breakpoints typically apply to a group of organisms rather than a specific species (21, 22), simplified presumptive bacterial identification schemes, based on manual phenotypic testing, were used instead of routine full identification. Many of these were based on the CLSI M35-A2 standard (23), but others were necessarily decisions based on local resources.

For example, frequent stockouts of the pyrolidonyl aminopeptidase (PYR) reagent made it difficult to distinguish *Enterococcus* sp. from α-hemolytic *Streptococcus* sp. For this important differentiation, we relied on optochin resistance and bile esculin positivity to define presumptive *Enterococcus* sp. when PYR reagent was unavailable. This is one among many procedures for which we had to develop identification schemes with alternatives to account for variability in reagent availability.

In other cases, importation of potentially expensive reagents was required for key processes. For instance, using discarded human plasma for coagulase testing instead of rabbit plasma, which was not available in Ethiopia, made it difficult to reliably make the critical distinction between *S. aureus* and other staphylococcal species. Thus, we relied on importation of a commercial kit (Pastorex™ Staph-Plus; Bio-Rad, Marnes-la-Coquette, France) to presumptively identify *S. aureus*.

A well-documented challenge faced by numerous laboratories in LRS is the lack of availability of sheep-blood agar (24). Expired human blood discarded by blood banks is often used instead of sheep blood, but yields unacceptably weak ß-hemolysis. This is especially problematic for the accurate identification of key pathogens like ß-hemolytic streptococci and *Listeria* sp. We tried to source a stable supply of sheep blood, by asking hospital administration to keep two healthy sheep on the hospital grounds for this purpose, and via agreements with local farmers, but neither option was successful. When human blood agar was used for blood-agar preparation, we stressed the importance of testing for catalase, and careful inspection of the hemolysis pattern using magnification and effective lighting. In addition, using human blood agar magnified the importance of routine Gram staining for confirming bacterial morphology, and of media quality control in general.

Finally, ambient temperature and humidity in tropical countries are frequently outside the normal operating conditions found in high-income settings, and for which the performance of most laboratory reagents and equipment are standardized. This has led for calls to produce “tropicalized” reagents and processes, as has been done in other industries (7). Fortunately, most of Ethiopia has a temperate climate due to its high elevation above sea level, and physical conditions did not prove to be a special challenge in the TASH laboratory.

### Training and Supervision of Laboratory Staff

Intervention studies for training highly qualified healthcare workers in low resources settings have repeatedly shown that providing well-formulated written guidelines in isolation is frequently unsuccessful, and that deployment of multiple simultaneous interventions may be more fruitful (25). Chief among these is direct supervision. To this end, mindful of the need for feasible demands on the time of prospective consulting supervisors, we budgeted a total of 50 visit-days/year from three microbiologists in our institution, usually in visits of 2 weeks' duration. Visits comprised a mix of formal training sessions with the technologists and daily plate rounds. There was a concerted effort to reach out to hospital clinicians via a series of invited lectures and clinical rounds on laboratory- and sepsis-related topics, and dedicated teaching sessions with the house staff. In the lab, the emphasis was on developing a culture of standard practices, promoting awareness and fostering the habit of using SOPs, and good work practices.

The visiting microbiologists reviewed workup of each specimen step-by-step during their periodic visits: from documentation of the specimen type, to reviewing of inoculation technique, to correlating Gram stain results with growth on plates, to interpreting identification and susceptibility testing results. Overall, we found that regular and repeated direct observations were the single most important action available for identifying knowledge and procedural gaps, and addressing them in a focused way.

The microbiologists were also essential in deciding which procedures were essential, and where deviations from standard practice could be tolerated without serious effects on quality. For example, because of shortages of media plates, technologists routinely inoculated up to four different specimens, one in each quadrant, of a single plate. This led to numerous contamination-related errors. Out of pragmatic need, accepting that up to 2 specimens could be inoculated per plate, or accepting the performance of some of the expired reagents if they passed quality control QC, were practice deviations we allowed.

Finally, we leveraged existing online resources to promote ongoing education of technologists. Each full-time technologist received a paid subscription to an online weekly proficiency testing program which included quizzes at the end of each module (26). Although it was originally intended as a proficiency-testing tool that could be followed from a distance, this programme proved popular among technologists, with noticeable improvements in knowledge over time.

### Implementation of a Practical Quality Systems Approach

Inaccurate laboratory results can harm patients, undermine confidence in the laboratory, and threaten downstream AMR control strategies. In all settings, it is crucial that sufficient resources and effort be invested in fostering a sustainable culture of quality assurance. Yet, achieving a broad-based commitment to quality from technologists and hospital administrators is especially challenging in settings where the value of clinical laboratories is already underappreciated, given the relatively invisible benefits of quality systems. Moreover, discussions about quality can be thwarted by fear of being blamed for errors.

At the hospital level, the Ethiopian Public Health Institute (EPHI) supported hospital administration to develop a quality manual aimed at providing the reference materials to satisfy basic requirements for SLIPTA (Stepwise laboratory quality improvement process toward accreditation) certification by the African Society of Laboratory Medicine (ASLM) (11). The manual was comprehensive, but did not translate into a culture of quality at the bench. The disconnect between written policies and practice led us to promote a shared-responsibility quality mindset, particularly the notion that identified problems should be seen as opportunities for learning and problem solving, rather than culpable mistakes. We also found that unrealistic expectations of perfection could be counterproductive and discourage quality improvement efforts.

Focusing on practical tasks, we gradually expanded existing QC to all analytical procedures in the bacteriology sector. A specific need that emerged upon development of several new QC procedures was the importance of clearly explaining the rationale for each of these steps to technologists, illustrated with concrete consequences of failed quality control tests and actions to take if/when these failures occurred. These explanations turned out to be critical: attempts to implement quality systems in the laboratory prior to this intervention were focused on administrative and operational aspects, and were removed from the day-to-day activities of the bacteriology sector. Introduction of a clear “quality-control calendar” in which weekly tasks were clearly scheduled was only moderately successful, for the simple reason that calendars and schedules are rarely used in the workplace in Ethiopia. Rather, we embedded quality control tasks within concrete pre-specified activities (e.g., QC after every media preparation; after opening a new reagent box, etc.) and ensured the action plans in case of QC failures were understood by all technologists.

Quality assurance (QA) activities included participation in EPHI's nascent external quality assessment programme, comprising a few bacterial isolates per year for which identification and antimicrobial susceptibility were assessed. We additionally performed internal quality assessments of the basic bacterial biochemical identification schemes by testing 20 randomly selected bacterial isolates with semi-automated API® identification strips (bioMérieux, France), twice yearly. Proficiency testing of technologists was done using the online Clinical Microbiology Proficiency Testing (CMPT) resource (26). In addition, we developed a tailor-made examination for TASH technologists that was administered once yearly, by the visiting microbiologists who served as external examiners. Audits of specific quality indicators were determined on the basis of needs identified during plate rounds or in discussion with laboratory users.

## Discussion

### Costs, Impact on Laboratory Utilization, and Downstream Effects of the Intervention

Our intervention involved only the bacteriology sector of the microbiology laboratory. Our initial investment was as follows. We received an in-kind donation from bioMérieux for a BacT/ALERT 3D 120 Combo instrument (bioMérieux, France), and secured an agreement with the local distributor that blood culture bottles for this instrument would be available on a regular basis. Beyond this, we budgeted and spent ~US$ 26,200 per year on blood culture bottles and consumables required for culture, bacterial identification, and antimicrobial susceptibility testing. Additional costs included ~US$ 1,900/year for an institution-wide subscription to a cumulative antibiogram tool accessible to end-users, as well as ~US$ 82 /technologist/year for access to the online proficiency testing resource described above. The costs for overhead and human resources in the laboratory were borne by the hospital itself. Moreover, these costs do not reflect the in-kind contribution of the academic microbiologists who provided ongoing oversight. In a memorandum of understanding, it was agreed by all parties that the running costs for the bacteriology laboratory would be assumed by the hospital after the end of the 4-year study period.

**Figure 1:**
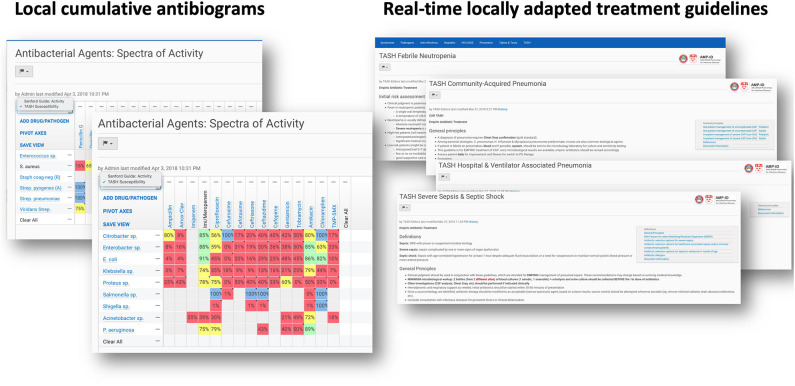
Screenshots of the commercially available subscription-based online resource on which we were able to insert cumulative antibiogram data as well as tailored clinical guidelines on antimicrobial use, informed by local susceptibility patterns (Sanford Guide with Stewardship Assist™; https://www.sanfordguide.com/stewardship-assist/). Accessible to all registered physicians at TASH via their smartphone.

Laboratory utilization increased markedly after implementation of the intervention (Table 2). The average total number of specimens increased from 15 per day to 75 /day within 18 months of initiation. For blood cultures, the average number of specimens received went from two per day to 45 per day within this period. This difference underestimates the impact on bloodstream infection diagnosis in the hospital, since the small number of blood culture requests prior to the intervention typically consisted in inadequate volumes of blood sent for manual processing. In contrast, standardized automated blood cultures and antimicrobial susceptibility testing by disc-diffusion are now provided routinely, with up to 50 blood culture specimens processed/day in the last 3 years. The laboratory further began supporting antimicrobial stewardship by generating regularly updated cumulative antibiograms (Figure 1). Finally, WHONET software (27, 28) was implemented in the laboratory with support from EPHI, in order to link data between institutional testing and national AMR surveillance. As a result, the bacteriology laboratory at TASH has contributed a significant portion of the carbapenem-resistance data submitted by EPHI in the last 2 years as part of the Global Antimicrobial Surveillance System (GLASS) (9). Overall, we observed that downstream laboratory-supported outputs created further demand for laboratory services.

**Table 2:** Selected metrics of laboratory utilization and laboratory-supported activities in Dec 2015- Jan 2016, prior to the laboratory strengthening intervention, and 18 months post-intervention. Laboratory utilization increased markedly in the post-implementation period.

	**Pre-intervention**	**Post-intervention**
Mean new blood cultures (*n*/day)	2	45
Mean total new specimens (*n*/day)	15	75
Cumulative antibiogram for hospital	No	Yes
Clinical diagnosis and treatment guidelines based on local susceptibility patterns	No	Yes
Antimicrobial stewardship activities	No	Yes

### Challenges and Mitigation Strategies

#### Overcoming Pre-analytical Obstacles in Hospital

In order to generate demand for appropriate microbiologic testing, we had to engage with experienced clinicians who were unaccustomed to access to reliable laboratory diagnostics. We did this through a series of clinician-focused hospital lectures on sepsis, septic shock, antimicrobial stewardship, and AMR, as well as topics requested by medical staff. For this activity, we suspect the fact that our team was comprised of specialists who were all certified in both clinical infectious diseases and in medical microbiology was an asset, though not essential. Although we believed didactic lectures to be required, it seems likely that even more impact on testing demand resulted from a related pharmacist-led antimicrobial stewardship intervention that leveraged medical chart audits and laboratory data to make weekly treatment recommendations for inpatients receiving antibiotics, described elsewhere (29, 30).

**Figure 2:**
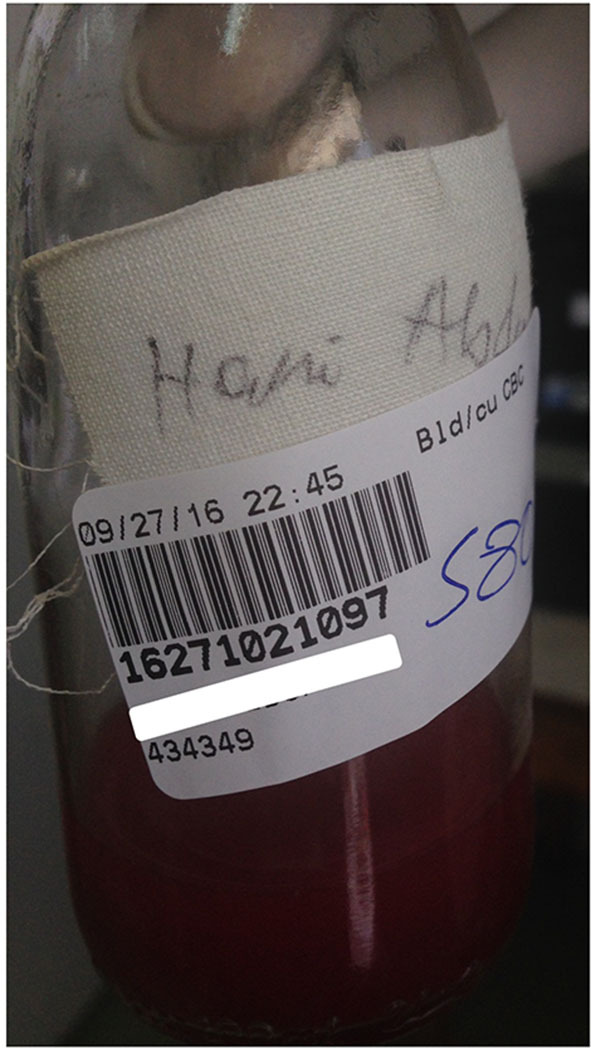
Photo illustrating several recurrent challenges in the pre-analytic phase of testing. A few mL of blood (i.e., inadequate volume) in a manual culture bottle (inappropriate collection device) without a requisition or any way to identify the requesting physician. The patient is identified only by name, without a unique identifier. The barcode shown was generated in the laboratory after a time-consuming investigation conclusively identified the patient and a requesting physician.

Once testing demand increased, recurrent challenges identified included specimens that were of poor quality, inadequately labeled, or for which the requested test was not indicated (Figure 2). One among many examples was the fact that despite having implemented automated blood cultures, the laboratory continued to receive grossly inadequate volumes of blood in manual culture broths for months, particularly from neonates and children, owing to misconceptions about testing accuracy and because of the lack of butterfly-type collection devices (BD, USA). In these cases, a strategy of clear communication, education, and persistence was generally successful, but would have been facilitated by the existence of a medically qualified microbiologist in the hospital.

#### Overcoming Post-analytical Obstacles in Hospital

A number of serious and sometimes perplexing obstacles in the post-analytic threatened the pertinence of our laboratory-strengthening intervention (Table 3). These included the absence of a functioning telephone, electronic medical record, or functional result printing service for the bacteriology sector. This made it difficult to report legible and clear bacteriology results to ordering physicians in a timely manner. At TASH, results are most frequently entered manually on the test requisition by the technologists, and placed in a folder that treating teams could collect. Further results are then copied in a laboratory log book which clinicians can consult on site. Prior to this intervention, no means of communicating urgent results such as positive blood cultures was available. We successfully implemented a policy of demanding the mobile phone number of the ordering physician on the test requisition, but attempts at consistently providing a printout of bacteriology results have so far not been successful.

**Table 3:** Key obstacles to the implementation of quality-assured clinical bacteriology laboratories. Many of the most difficult challenges are outside the laboratory and its control.

**Challenge encountered**	**Solution attempted or proposed**
**Overall quality assurance**	
Achieving a broad-based commitment to quality	• Sustained engagement with clinicians and hospital administration to promote the relevance of the laboratory. • Leveraging key colleagues as “champions” of the laboratory. • Focusing on achievable goals, and avoiding unrealistic expectations of perfection. • Promoting a shared-responsibility quality mindset, and the objective of constant improvement.
**Pre-analytical phase**	
Engaging and generating demand from clinicians	• Series of clinician-focused hospital lectures. • Pharmacist-led antimicrobial stewardship intervention for inpatients receiving antibiotics, described elsewhere (29).
Poor-quality specimen Wrong test ordered	• Communication, education, and persistence.
Inappropriate transport conditions	• Would be facilitated by the existence of a medically qualified microbiologist in the hospital.
Improperly labeled specimens Absent, incomplete, incomprehensible requisitions	• Technologists provided blood culture bottles only after requesting physicians supplied a properly filled requisition. • Verifying labeling on specimens on arrival.
**Analytical phase (in laboratory)**	
Adapting standard operating procedures (SOP)	• Using best-practices for SOP in LRS (20), including straightforward language, short sentences, a legible font and size, and avoiding acronyms. • AVOIDING pictograms, illustrations, or figures as a *replacement* of plain language. • AVOIDING calendar-based QC schedule. Instead, embedding QC tasks within concrete pre-specified activities (e.g., QC after opening a new reagent box).
Standardizing practice	• Regular and repeated direct observations were the single most useful action available for identifying knowledge and procedural gaps, and for promoting reference to SOPs.
Availability of pyrolidonyl aminopeptidase (PYR) for identification of *Enterococcus* sp.	• In the absence of PYR reagent, we relied on bile esculin (BE) hydrolysis to differentiate *Enterococcus* sp. from the majority of alpha-hemolytic Viridans Group *Streptococcus* sp. other than *S. bovis*. Optochin susceptibility was determined in order to identify *S. pneumoniae* among *Streptococcus* sp. that may hydrolyse BE. • Pathogens potentially misidentified as *Enterococcus* sp. using this second-line approach include *S. mutans, S. salivarius*, and *S. suis*, but these are infrequent blood-borne pathogens and would be treated by regimens directed at *Enterococcus* sp.
Availability of rabbit plasma for coagulase testing	• We relied on importation of a commercial kit (Pastorex™ Staph-Plus; Bio-Rad, Marnes-la-Coquette, France) to presumptively identify *S. aureus*. • Alternatively, human plasma discarded from blood banks could be used instead of rabbit plasma or commercial kit, but it needed to pass QC using an appropriate *S. aureus* strain.
Availability of Mueller-Hinton blood agar for AST of fastidious bacteria	• Use of chocolate agar was permitted as long as QC testing of antimicrobial discs using representative bacterial strains yielded expected results.
Availability of sheep-blood agar	• Hospital administration unwilling to keep two healthy sheep on the hospital grounds for this purpose. • Approached local farmers to bleed their sheep, but could not agree on reimbursement and logistics. • When human blood agar was used for blood-agar preparation, we stressed the importance of testing for catalase, and careful inspection of the hemolysis pattern using magnification and effective lighting. In addition, using human blood agar magnified the importance of routine Gram staining for confirming bacterial morphology, and of media quality control in general.
Complexity of bacterial identification	• Simplified presumptive bacterial identification schemes, based on manual phenotypic testing, were used instead of routine full identification.
Inadequate space	• Few short-term solutions.
**Post-analytical phase**	
No telephone system in hospita No way to reach physician for critical results	• Successfully implemented a policy of demanding the mobile phone number of the ordering physician on the test requisition.
Inadequate laboratory information system	• Implemented WHONET software (27, 28) with support from EPHI, in order to link data between institutional testing and national AMR surveillance. • Unfortunately, WHONET is not capable of interfacing with the regular laboratory information system in the laboratory. • Unfortunately, WHONET is not capable of interfacing with in-hospital electronic medical record systems.
No in-hospital electronic medical record	• We relied on a written ledger that was consulted on a daily basis by ordering physicians. • Efforts to provide printouts of WHONET entries in lieu of this ledger were thwarted by consistent lack of consumables (paper, toner) and controversy about the need for this step.
	• We provided all registered physicians with electronic access to a commercially available online resource on which we could load cumulative antibiogram data as well as tailored clinical guidelines on antimicrobial use, informed by local susceptibility patterns (Sanford Guide with Stewardship Assist™; https://www.sanfordguide.com/stewardship-assist/.
Data analysis responsibility	• Ongoing data analysis for quality assurance remains a challenge without external support.
Power/training differential between technologists and laboratory clients	• The promotion of mutual respect through lectures and open communication was helpful.
**Challenges outside the testing cycle**	
Overcoming local supply and foreign currency exchange issues	• See text.
Engagement from hospital & university community toward importance of lab sector	
Attractiveness of microbiology as a career path, given lack of recognized specialty in LRS	
Attractiveness of Infectious Diseases as a career given lower revenue compared to procedural specialties.	

*SOP denotes standard operating procedures; PYR, pyrolidonyl aminopeptidase; AMR, antimicrobial resistance; AST, antimicrobial susceptibility testing; LRS, low resource settings; QC, quality control; BE, bile esculin*.

The lack a hospital-wide electronic medical record (EMR) capable of interfacing with the laboratory information system remains an outstanding problem. However, we tried to palliate this issue by leveraging the fact that almost all hospital employees have mobile smartphones. Although we were not in a position to create an EMR, we provided all registered physicians at TASH with electronic access to a commercially available online resource on which we could load cumulative antibiogram data as well as tailored clinical guidelines on antimicrobial use, informed by local susceptibility patterns (Sanford Guide with Stewardship Assist™; https://www.sanfordguide.com/stewardship-assist/). Using this tool, which requires an institutional subscription, clinical staff could circumvent infrastructural barriers. Of course, this required access to Wi-Fi in the hospital and adoption by clinicians, both of which proved to be only partial successes. An unexpected source of technical difficulties was the widespread use of internet proxy servers, from which institutional accounts could not be accessed. This thwarted easy adoption of smart-phone-based access.

#### Overcoming Obstacles Outside Hospital

By far the most vexing issue that threatened sustainability of our intervention was securing a stable local supply chain for essential laboratory reagents. In theory, the government ensures a national laboratory supply chain of quality assured pharmaceuticals and laboratory reagents through the national Pharmaceutical Supply Agency (EPSA), which procures items through a bidding process from a list of suppliers. In practice, this agency faces significant challenges in procuring and delivering adequate supplies of specific reagents to specific institutions, resulting in frequent stock-outs. Once delivered, a significant proportion of specific items purchased through this route (for e.g., antibiotic discs) fail quality control testing—leaving us with little alternative other than purchasing critical reagents from selected manufacturers.

In addition to regulatory obstacles that force laboratories to go through the single national supplier, private purchases are themselves thwarted by profound foreign exchange distortions that make it virtually impossible for buyers in Ethiopia to purchase goods on the international market. We palliated this issue through direct purchase and importation from Canada, but this is clearly not sustainable in the absence of outside collaborators.

Engagement of the hospital & university community about the importance of a healthy laboratory sector remains important. In addition to the necessary provision of budgetary allowances, this impacts on the attractiveness of microbiology as a career path, for without institutional prestige or prospects for advancement, few candidates are likely to enter the field. Similarly, recruitment to the related medical subspecialty speciality of clinical infectious diseases is often complicated by a lower expected revenue compared to more procedural specialties. These are issues that require system-level solutions that reflect adequate prioritization of clinical bacteriology in public health agendas.

### Impact on Laboratory Quality—External Audits and Accreditation Experience

An independent audit of the bacteriology sector at TASH from the American Society of Microbiology in October 2017 yielded a 4-star rating (of a possible five stars), citing marked improvements in the analytic phase processes and in quality control. On the basis of this result and our own self-evaluations, we requested an audit from ASLM for SLIPTA certification in late 2018 (11). The final result from this audit was worse than expected (189/265 points; two stars), as the emphasis was predominantly on structural and administrative processes (e.g., Management Reviews, for which TASH received 1/14 points) with disappointingly few points given for analytical and quality processes performed in the laboratory. While this had a somewhat demoralizing effect on laboratory staff, the experience did suggest that professional societies, who have deep content expertise, may offer especially pertinent external feedback in the early stages of laboratory quality improvement initiatives.

## Where Do We Go From Here?

The present-day crisis of widespread antimicrobial resistance in LRS means that the clinical bacteriology sector can no longer be regarded as optional. Although technological innovations are needed, there are no practical alternatives to current techniques for phenotypic antimicrobial susceptibility testing in the short-term. Using a relatively low-intensity intervention based on existing training tools and accreditation schemes, we demonstrate that establishment of reasonable-quality clinical bacteriology is within reach. Yet, to show that a laboratory strengthening intervention is feasible does not necessarily imply that is sustainable, even if it achieves a benchmark of cost-effectiveness. Sustainability is a different beast. It depends on local priorities, which makes it more difficult to safeguard.

We noted in the introduction of this *Community Case Study* that we sought to demonstrate the feasibility of a practical bundle of laboratory-strengthening interventions. We did this to help bridge the gap between high-level recommendations from WHO and governments, and the poor availability of clinical bacteriology laboratories in sub-Saharan Africa. A key lesson learnt in the course of our work is that in addition to sound recommendations and demonstrated feasibility, priorities of the organizational leadership must coincide with them to yield sustainability.

To this end, it is vital that Ministries of Health and health administrators urgently align their priorities to implement a whole systems quality approach, rather than address AMR as an issue to be solved through a set of limited actions. Diagnostic bacteriology laboratories should receive adequate, secure and sustained budgetary support to ensure that they can routinely provide quality-assured testing necessary for AMR containment. This includes the need for upgrading facilities and for staff to be trained in the new techniques necessary for effective AMR diagnostics. Crucially, medical microbiology should be a recognized medical specialty in each country and be prioritized in order to create a pool of experts capable of liaising with clinicians and infection control practitioners to coordinate containment strategies for AMR.

## Data Availability Statement

The datasets generated for this study are available on request to the corresponding author.

## Ethics Statement

The studies involving human participants were reviewed and approved by Research Ethics Board of the RI-MUHC. Written informed consent for participation was not required for this study in accordance with the national legislation and the institutional requirements.

## Author Contributions

CY coordinated the writing of the manuscript. CY and MS wrote a first draft. All authors reviewed the manuscript and added intellectual content.

## Conflict of Interest

In-kind support from bioMérieux was provided via an investigator-initiated research grant to MS and CY. CY holds a Chercheur-boursier clinicien career award from the Fonds de recherche du Québec-Santé (FRQS). The remaining authors declare that they have no commercial or financial relationships that could be construed as a potential conflict of interest.
