# Supplementary material for: A Feasible Laboratory-Strengthening Intervention Yielding a Sustainable Clinical Bacteriology Sector to Support Antimicrobial Stewardship in a Large Referral Hospital in Ethiopia
Source: Front Public Health. 2020 Jun 23;8:258. doi: 10.3389/fpubh.2020.00258 (PMC7325602; doi:10.3389/fpubh.2020.00258)
Supplement: Supplementary file 1 [file Data_Sheet_1.PDF]

AMP-ID
AAU-McGill Partnership for Infectious Diseases

Tikur Anbessa Specialized Hospital

Microbiology Laboratory

STANDARD OPERATING PROCEDURE

ANTIMICROBIAL SUSCEPTIBILITY TESTING (AST) USING KIRBY-BAUER DISK DIFFUSION

Drafted by M. Semret, Jan 12, 2016

1. PRINCIPLE AND PURPOSE

1.1. The principle of the disk diffusion test is an *in vitro* method for quantitative antimicrobial susceptibility testing whereby a preformed antimicrobial gradient from an antibiotic-impregnated disk diffuses into the Mueller-Hinton agar inoculated with the organism to be tested.

1.2. The purpose of the Kirby-Bauer disk diffusion susceptibility test is to determine the sensitivity or resistance of a pathogenic organism to various antimicrobial drugs.

1.3. A standardized inoculum (0.5 McFarland) of the pathogenic organism is swabbed onto a Mueller Hinton agar (MHA) plate. Filter paper disks impregnated each with a known concentration of an antimicrobial substance are placed onto the agar. After 18-24 hours of incubation, the diameter of the zone of inhibition is measured around each disk.

1.4. By measuring zone sizes and interpreting them according to the Clinical Laboratory Standards Institute (CLSI) Performance Standards for Antimicrobial Susceptibility Testing, a qualitative report is obtained, meeting the criteria of susceptible (S), intermediate (I), or resistant (R).

1.5. The results of the Kirby-Bauer disk diffusion susceptibility test is performed not only to assist physicians in selecting the proper treatment option but also to monitor antibiotic resistance patterns.

2. REAGENTS AND SUPPLIES

2.1. Media and reagents

- Mueller Hinton Agar plates with or without 5% blood stored at 2-8°C.
- GC agar base with defined supplements stored at 2-8°C.
- Antimicrobial disks stored at -14°C to 8°C.
- Beta-lactamase class antimicrobial agents should be stored at -14°C or below in a frost free freezer as they are very labile.

2.1.1. Supplies

- Sterile cotton tipped swabs
- Sterile plastic pipettes
- McFarland Turbidity Standards
- Forceps
- Ruler or sliding caliper

- Vortex mixer
- Ambient air and CO_2 incubators

3. METHOD

3.1. Preparation of innoculum

- Use direct colony suspension inoculum
    - Using a sterile swab, select 4-5 well isolated colonies of similar morphology and inoculate into Mueller Hinton Broth or sterile saline.
    - Adjust the turbidity of the suspension to match a 0.5 McFarland.
    - Use the suspension within 15 minutes of preparation.

3.2. Procedure

- Bring all media and disks to room temperature before use.
- Dip a sterile swab into the inoculum, when removing the swab, rotate the swab against the side of the tube while applying pressure to remove excess liquid from the swab prior to inoculating the plate.

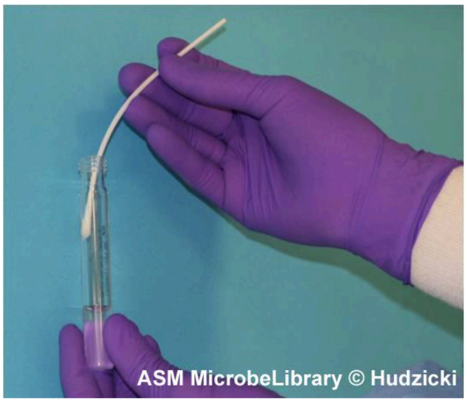

- Inoculate the plate by streaking the swab using firm pressure over the entire agar surface. Rotate the plate 60° and streak the entire plate again. Repeat this action a third time. This ensures an even distribution of inoculum that will result in a confluent lawn of growth.

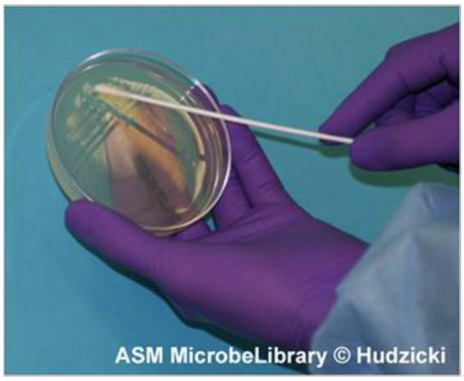

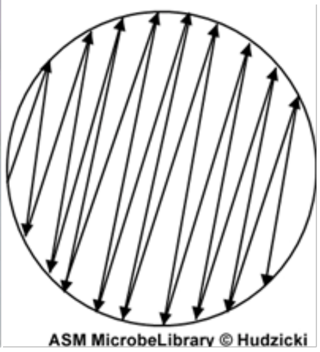

- After streaking the Mueller-Hinton as described, rim the plate with the swab by running the swab around the edge of the entire plate to pick up excessive inoculum that may have splashed near the edge. DO NOT REENTER THE PLATE with the swab as it may lead to contamination.

3.3. Application of the disks

- Using sterile forceps, remove a disk from the disk dispenser and place it directly on the MHA. Apply gentle pressure with the forceps to ensure complete contact of the disk agar.
- DO NOT dispense the disks onto another surface before placing them on the agar.
- Use up to 5 disks on a 100mm plate

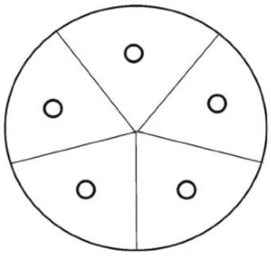

- Use up to 12 disks on a 150mm plate

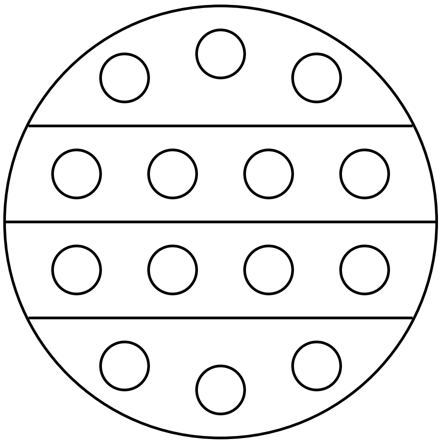

3.4. Incubation
- Invert plates and incubate them within 15 minutes of disk application.
- Place in appropriate environment described in AST laboratory bench aid (see Annex 1).

3.5. Reading plates and Recording Results
- Read plate after 18 hours (maximum 24 hours).

- Read plates only if there is confluent or nearly confluent growth.
- Measure the zone sizes to the nearest millimeter using a ruler or a caliper.
  - When measuring zone sizes, always round up to the nearest millimetesr
  - Refer the AST laboratory bench aid to measure and interpret zone sizes.
- Record the actual zone sizes on the work-card (not just the interpretation).
- Growth up to the disk should be reported as 0mm.
- For *Proteus*, disregard the swarming and measure the edge of obvious inhibition.

4. REPORTING RESULTS

- Report the results of the Kirby-Bauer disk diffusion susceptibility as susceptible (S), intermediate (I) or resistant (R).
- Do not report the zone sizes to physicians.

5. LIMITATIONS

5.1. Characteristic of isolate

- This method is standardized only for rapidly growing aerobes, including *Enterobacteriaceae. Pseudomonas aeroginosa, Acinetobacter* spp., *Staphylococcus* spp., and *Enterococcus* spp..
- Modifications have been done for fastidious organisms (*Streptococcus* spp., *Haemophilus* spp., and *N. gonorrhea*).
- For other organisms not mentioned above, an MIC test (broth microdilution) should be done.

5.2. Numerous factors can affect results

- inoculum size
- rate of growth
- incubation time/atmosphere
- viability of disks

6. References

McGill University SOP for Kirby-Bauer Disk Diffusion Test written by Toula Nikolaou (2012).
