# Supplementary material for: A Feasible Laboratory-Strengthening Intervention Yielding a Sustainable Clinical Bacteriology Sector to Support Antimicrobial Stewardship in a Large Referral Hospital in Ethiopia
Source: Front Public Health. 2020 Jun 23;8:258. doi: 10.3389/fpubh.2020.00258 (PMC7325602; doi:10.3389/fpubh.2020.00258)
Supplement: Supplementary file 2 [file Data_Sheet_2.PDF]

AMP-ID
AAU-McGill Partnership for Infectious Diseases

Tikur Anbessa Specialized Hospital

Microbiology Laboratory

STANDARD OPERATING PROCEDURE

BACTERIAL CULTURE OF CSF

Drafted by M. Semret, Jan 12, 2016

1. PURPOSE AND PRINCIPLE

1.1. The purpose of this protocol is to describe the procedure for the culture of cerebrospinal fluid (CSF).
1.2. Bacterial meningitis is a life-threatening infections that requires emergent analysis of the CSF to guide treatment.
1.3. The most common pathogens are:
- *Neisseria meningitis*
- *Streptococcus pneumonia*
- *Haemophilus influenza* type B
1.4. In neonates, the most common pathogens are:
- *Enterobacteriaceae* (*E. coli* is most common)
- Group B *Streptococcus*
- *Listeria monocytogenes*
1.5. In immunocompromised, all of the above pathogens can be found in addition to fungal and mycobacterial organisms.

2. CLINICAL SAMPLE

2.1. Specimen types include the following:
- Lumbar puncture spinal fluid
- Central nervous system shunt fluid
- External ventricular drainage fluid

2.2. CSF should be collected in a sterile tube.

2.3. Any volume is accepted, but ideally there should be at least 1 mL for culture.

3. RECEPTION

3.1. CSF SPECIMENS MUST BE PROCESSED IMMEDIATELY UPON RECEPTION IN THE MICROBIOLOGY LABORATORY.
3.2. THE SPECIMENS MUST NOT BE REFRIGERATED AT ANY TIME.
3.3. **PROCESSING OF ALL CSF SAMPLES MUST BE DONE IN A BIOLOGICAL SAFETY CABINET.**

3.4. Acceptation criteria

- All specimens are accepted.
- If there has been delay in transport or inadvertent refrigeration, the order must be processed and the divergence from the processing protocol must be recorded.
- If the specimen is mislabeled, it must be processed and the sample origin must be tracked.

3.5. Rejection criteria

- No specimen will be rejected

4. EQUIPMENT AND MATERIAL

4.1. Equipment

- Sterile glass slides
- Gram stain reagents
- Centrifuge
- Vortex mixer
- Sterile pipettes

4.2. Material

- 5% Sheep Blood agar (SBA)
- Chocolate agar (CHOC)
- MacConkey agar (MAC)
- Nutrient Broth

5. METHOD

5.1. Sample preparation

ALL SAMPLE PROCESSING MUST BE PERFORMED UNDER THE BIOLGICAL SAFETY CABINET until *N. meningitis* has been ruled out.

- Record the appearance of the CSF (clear, bloody, cloudy, etc.) and approximate volume of CSF received.

<u>If less than (≤1 mL) received</u>

- Do not centrifuge
- Vortex specimen in biological safety cabinet with the CAP ON
- Proceed with Gram stain.

If greater than (>1 mL) received

- Centrifuge specimen for 5 minutes at 3,000g.
- Aspirate the supernatant with a sterile pipette leaving 1mL of specimen in the tube. Keep the supernatant in a sterile tube in the fridge at 4°C until the final report is issued.
- It is CRTITICAL to vortex the SEDIMENT vigorously with the CAP ON for 30 seconds to re-suspend the pellet.
- Proceed with Gram stain.

5.2. Microscopy

- Quantitate white blood cells and organisms as per Gram stain bench aid.
- Interpret all CSF Gram stains immediately and report all positive findings immediately to requesting physician (see Reporting section).

5.3. Inoculation & Incubation

Aspirate from the bottom of the collection tube and inoculate 3 drops to the following media

- BAP
- CHOC
- MAC ONLY IF GRAM-NEGATIVE RODS SEEN ON GRAM STAIN
- NUTRIENT BROTH

If less than 1mL of CSF is received and there is insufficient sample for all the plates:

- Place 1 drop on a CHOC agar
- Using the same pipette, add 0.5mL of Nutrient Broth to the specimen tube.
- From the Nutrient Broth, inoculate a BAP (and a MAC if GNR seen on Gram)

At 24 hours:

- Read all plates
- Inoculate broth onto a CHOC and incubate in CO_2 at 35°C.

At 48 hours:

- Read all plates, including CHOC from the broth.

At 72 hours:

- Read all plates, if no growth, discard them all.

6. IDENTIFICATION

6.1. Organisms to identify:

- ALL ORGANISMS should be identified according to appropriate identification SOP.
- N.B. Coagulase negative *Staphylococcus* and *Corynebacterium* spp. may be true pathogens, therefore their presence must be reported. The clinical significance of the organism is to be determined by the treating team.

6.2. Antibiogram:

- AST is to be done on as per the AST bench aid.

7. RESULTS REPORTING

7.1. Critical results

- ALL POSITIVE GRAM STAINS ARE CONSIDERED TO BE CRITICAL RESULTS
- The requesting physician/team must be informed immediately and this communication must be documented.
- Gram stain reports should be documented on the work-card as well as the log book as the organism may fail to grow in culture.

7.2. Preliminary report

- If there is no growth on the plates or if the broth is not turbid at 24 hours, a preliminary report: 'No growth at 24 hours, final report to follow', shoulde be recorded.

7.3. Final report

- If positive growth, report all organisms with susceptibilities as appropriate.
- If no growth, report: 'No growth at 72 hours'.

8. LIMITATIONS

- This procedure does not describe the isolation and culture of fungal elements.
- A 72-hour total incubation period has been chosen in order to provide the highest yield in terms of recovering an organism weighed against the increasing likelihood of contamination with prolonged incubation times.
- Should the original Gram-stain be suggestive of *Listeria*-like morphology and fails to grow with the above described procedure, a Microbiologist or senior technician should be consulted in order to set up a cold enrichment technique.

9. REFERENCES

9.1. McGill University SOP for CSF Culture, written by Dr. Earl Rubin (2004)
9.2. Clinical Microbiology Procedures Handbook, 3rd Edition, 2007, Editor in Chief: Henry D. Isenberg
